# Effect of Nanoparticles on Modified Screen Printed Inhibition Superoxide Dismutase Electrodes for Aluminum

**DOI:** 10.3390/s16101588

**Published:** 2016-09-26

**Authors:** Miriam Barquero-Quirós, María Julia Arcos-Martínez

**Affiliations:** 1 Department of Chemistry, University of Costa Rica, CELEQ, San Pedro de Montes de Oca, San José 11501-2060, Costa Rica; 2 Department of Chemistry, Faculty of Sciences, University of Burgos, Plaza Misael Bañuelos s/n, Burgos 09001, Spain; jarcos@ubu.es

**Keywords:** superoxide dismutase biosensor, aluminum, tetrathiofulvalene, screen-printed electrodes, nanoparticles

## Abstract

A novel amperometric biosensor for the determination of Al(III) based on the inhibition of the enzyme superoxide dismutase has been developed. The oxidation signal of epinephrine substrate was affected by the presence of Al(III) ions leading to a decrease in its amperometric current. The immobilization of the enzyme was performed with glutaraldehyde on screen-printed carbon electrodes modifiedwith tetrathiofulvalene (TTF) and different types ofnanoparticles. Nanoparticles of gold, platinum, rhodium and palladium were deposited on screen printed carbon electrodes by means of two electrochemical procedures. Nanoparticles were characterized trough scanning electronic microscopy, X-rays fluorescence, and atomic force microscopy. Palladium nanoparticles showed lower atomic force microscopy parameters and higher slope of aluminum calibration curves and were selected to perform sensor validation. The developed biosensor has a detection limit of 2.0 ± 0.2 μM for Al(III), with a reproducibility of 7.9% (n = 5). Recovery of standard reference material spiked to buffer solution was 103.8% with a relative standard deviation of 4.8% (n = 5). Recovery of tap water spiked with the standard reference material was 100.5 with a relative standard deviation of 3.4% (n = 3). The study of interfering ions has also been carried out.

## 1. Introduction

Aluminum toxicity has been shown in vivo and in vitro, but complexity of its interactions with human organism makes it very difficult to assign the responsibility in Alzheimer’s disease. It can be considered as the combined effect of oxidant action, participation on amyloid cascade, neuronal degeneration [1] and accumulation in neurofibrillary tangles. Aluminum presence favors T protein link trough phosphate bridge [2] and alters homeostatic ion equilibrium [3], and it has shown a strong effect on reactive oxygen species (ROS) production on living organisms, due to iron accumulation in oxidative stress [4]. Markedly, ROS impaired enzymes such as superoxide dismutase (SOD) and catalase react with radical species such as O_2_•^−^, OH•^−^, and ONNO•^−^. Although O_2_•^−^ radical is not so reactive itself, in the presence of Fe^2+^, Fenton reaction can turn it into hydroxyl radical, which is the most potent radical. These findings show that O_2_•^−^ is involved in cellular damage. Biosensors have employed superoxide dismutase (SOD), an enzyme that scavenges superoxide to measure superoxide anion accordingly with the reactions [5]:

Cu^2+^ − SOD + O_2_•^−^ → Cu^1+^ − SOD + O_2_

Cu^1+^ − SOD + O_2_•^−^ + 2H^+^ → Cu^2+^ − SOD + H_2_O_2_

O_2_•^−^ affects cytochrome c (Cyt c) by oxidizing Fe^2+^ to Fe^3+^ and reducing itself to H_2_O_2_. O_2_•^−^ radical levels were found to be elevated in homogenized cancerous brain tissue compared to normal human brain tissue [6]. O_2_•^−^ is formed in living biological systems by the donation of an electron to molecular oxygen, through oxidation of semiquinone-type radicals formed in the mitochondrial electron transport chain. In its presence, free radical scavengers, enzymes such as superoxide dismutase and glutathione peroxidase (GSH-Px), decrease their antioxidant status, and lipid peroxide levels are increased.

Aluminum administration to laboratory animals induces SOD dysfunction and damage on target organs [7,8,9,10]. It has been found that zinc [11], selenium [12] and therapies used against Alzheimer’s disease [13,14,15,16] have a protective role against aluminum induced toxicity, improving SOD function. Aluminum presence in humans is associated with oxidative stress [17,18].

Biosensors constructed with screen printed electrodes (SPCE) offer low detection limits, easier assembly of metallic nanoparticles (NPs), good reproducibility, low contamination and excellent biocompatibility with enzymes and antibodies [18]. The metal NPs’ small size, high mechanical strength and high chemical and thermal stability allow them, when acting as enzyme-carrier materials, to improve the efficiency of immobilized enzymes, facilitating reaction kinetics, and supplying a larger surface area, leading to higher enzyme loading per unit mass of particles. This fact allows achieving enhanced device sensitivity and reduced mass transfer resistance [19]. Besides, NPs increased electric conductivity and electron transfer between redox enzyme center and electrode [20,21].

Different types of NPs such as metal, metal oxides, semiconductors, polymers and composite-metal NPs have been used to assembly miniaturized electrochemical sensors and biosensors. Gold NPs (AuNPs), due to their unique properties, relatively low cost and ease of preparation, are the most used in many biochemical applications [22,23]. AuNPs can be synthesized by different chemical methods and applied to electrochemical detection of As(III) [24]. AuNPs use in amperometric biosensors and electrochemical techniques enhances detection sensitivity [25,26,27]. 

Due to their inertness, platinum NPs (PtNPs) are the principal metal NPs alternative for anodic current measurement and have been applied to formaldehyde [28], neurotransmitters [29], glucose [30], uric acid [31], and As(III) determination [32]. 

Glassy carbon electrodes modified with palladium nanoparticles (PdNPs) have been applied to catecholamines determination [33]. PtNPs and PdNPs/methylthiophene (PMT) sensors have been applied to dopamine (DA) and AA determination [34].

Rhodium NPs’ (RhNPs) main application has been as precursors for the preparation of catalytics [35,36] and for catalysis and sensing of cytochrome c [37] and H_2_O_2_ [38], as well as for biosensing of α-ketoglutarate [39]. 

Due to sensitivity and specificity joint benefits produced by modified SPCEs with metallic NPs and enzymes, this research work was conducted with the goal to compare the effect of NPs of Au, Pt, Pd and Rh deposited by distinct electrochemical procedures on sensitivity of amperometric inhibition SOD by aluminum, with TTF as mediator using epinephrine (EPI) as substrate.

Most developed biosensors that immobilize SOD enzyme in a carrageenan gel are based on H_2_O_2_ amperometric detection [40] and were successfully applied as a tool for antioxidant capacity assessment to evaluate red and white wines [41], fresh herbs and fruits, olives, tea [42,43,44], algae [45], phytoterapeutic preparations [46], drugs containing salicylic and as corbic acid, and β-carotene [47,40]. This developed biosensor enables measurement of antioxidant capacity of healthy and diseased tissues in vitro [40], and was also applied to determination of total antioxidant capacity of berries [48].

The others SOD based biosensors that employ modified solid electrodes are shown in Table 1.

Taking into account that TTF allows the rapid electron transfer between SOD and electrode surface can be carried out at lower potential [57], and that pro-oxidant activity of aluminum inhibits SOD activity [58], this study indicates that Al(III) SOD inhibition can be performed at lower potential compared to other aluminum enzymatic determinations. It was shown for the first time using SPC_TTF_Es that Al(III) inhibits SOD enzyme linked with Alzheimer’s disease at low concentrations.

## 2. Materials and Methods 

### 2.1. Reagents

All solutions were prepared with purified water supplied by TKA Gen Pure, inverse osmosis, with a UV lamp irradiation system.

SOD enzyme (30 KU), EPI, bovine serum albumine (BSA), glutaraldehyde (GA) and hydrogen tetrachloroaurate (III) trihydrate (HAuCl_4_) were obtained from Sigma-Aldrich (Sigma-Aldrich), Steinheim, Germany). 

Solutions of platinum, rhodium and palladium 0.1 mM were prepared from ICP solutions of 1000 mg/L (Merck, Darmstad, Germany).

Titrisol solutions from (Merck, Darmstad, Germany)were used to prepare stock standard solutions of Al(III), Cu(II), Fe(III), Sn(II), Zn(II), Co(II), Ni(II), Se(IV) Cr(III), Cd(II), Pb(II), W(VI) and V(V). Mo(VI) and Ca(II) solutions were obtained from Inorganic Ventures (Lakewood, NJ, USA). As(V) and Hg(II) solutions were prepared from Atomic Spectroscopy Standards solutions (Perkin Elmer Co, Whaltham, MA, USA).

Al(III) solutions used for spike were prepared from High Purity Standards (Charleston, SC, USA) confirmed against standard reference material SRM 3101.

Britton Robinson (BR) supporting electrolyte solutions were prepared as usual with boric, phosphoric and acetic acids (Merck, Darmstadt, Germany), and the required pH was obtained by adjusting with NaOH solution (Suprapur, Merck, Darmstadt, Germany).

Several inks were used in the fabrication of SPEs, namely Electrodag PF-407 A (carbon ink), Electrodag 6037 SS (silver/silver chloride ink) and Electrodag 452 SS (dielectric ink) supplied by Acheson Colloiden (Acheson Colloiden, Scheemda, The Netherlands).

The working electrode ink was prepared by thoroughly mixing carbon ink with tetrathiofulvalene (C_TTF_) 5%. TTF was obtained from Acros Organics (Acros Organics, Geel, Belgium).

### 2.2. Equipment

An electrochemical system Autolab PGSTAT Echo Chemie128 N with GPS software was used to record electrochemical measurements (Echo Chemie, Utrech, The Netherlands). 

All pH values were adjusted with a pHmeter (Mettler Toledo, Schwerzenbach, Switzerland). 

A S-3700 Hitachi was used to perform scanning electronic microscopy (SEM) of SPCEs. An IXRF Systems model 550*i* was used to obtain spectra of elements on the SPCE. Atomic force microscopy (AFM) parameters and images were obtained with a NanoScopeQuadrex Digital Instruments Veeco Metrology Group.

#### SPC_TTF_Es Construction

**Figure 1:**
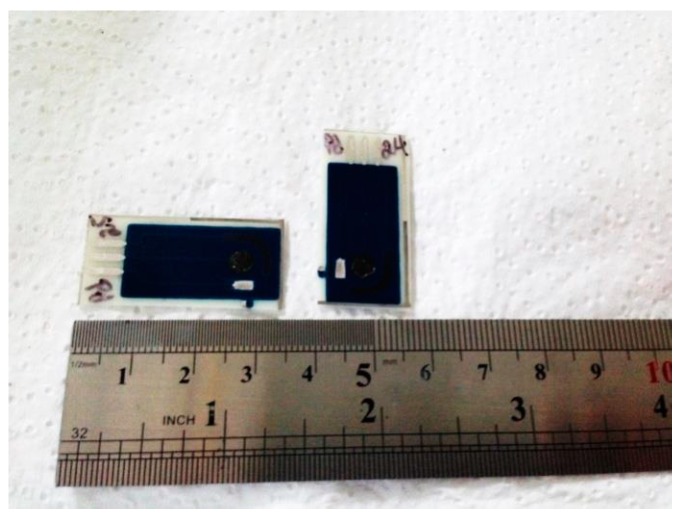
Screen printed electrodic system used.

SPC_TTF_Es were homemade built using a DEK 248 printing machine (DEK, Weymouth, UK) using polyester screens with appropriate stencil designs mounted at 45° to the printer stroke. These transducers consisted of three screen-printed electrodes deposited onto polyethylene terephthalate films (HiFi Industrial Film, Dardilly, France). The different inks were screen-printed and cured according to the manufacturer’s specifications. The working electrode ink was prepared by thoroughly mixing carbon ink with TTF (5% *v*/*w*) and immediately screen-printed. One electrode is shownin (Figure 1).

### 2.3. Nanoparticles Electrodeposition Methods

SPC_TTF_Es modification with nanoparticles (NPs/SPC_TTF_Es) was carried out by both controlled potentialand cyclic voltammetry scan methods.

*(A) Metal plating* was carried at two different potentials namely +0.3 and +0.18 V, in a quartz cell containing Au(III), Pt(IV), Rh(IV) or Pd(IV) solutions (0.1 mM) in H_2_SO_4_ (0.5 M) [24]. Following electrodeposition process, the NPs/SPC_TTF_Es was removed from platting solution, rinsed with purified water and wiped carefully.

*(B) Cyclic voltammetry* deposition was performed doing a set of seven successive voltammetric scans between +1.0 and −0.2 Vin a quartz cell containing Au(III), Pt(IV), Rh(IV) or Pd(IV) (0.1 mM) in H_2_SO_4_ (0.5 M) [59]. Electrodes were prepared by setting two cyclic voltammetric conditions namely CV1 and CV2.

CV1: delay time 60 s, step potential 0.0150 V, scan rate 0.050 V/s.

CV2: delay time 120 s, step potential 0.025 V, scan rate 0.100 V/s.

After nanoparticles deposition, the electrode was rinsed with purified water and wiped carefully.

### 2.4. SOD Enzyme Immobilization onto AuNPs//SPC_TTF_ Es

Enzyme was immobilized by crosslinking polymerization with glutaraldehyde [60] on the surfaces of AuNPs/SPC_TTF_Es, PtNPs/SPC_TTF_Es, PdNPs/SPC_TTF_Es, and RhNPs/SPC_TTF_Es. To carry out the immobilization procedure, superoxide dismutase enzyme solution was prepared by dissolving enzyme in Britton Robinson buffer at pH 7.0. To avoid loss of enzymatic activity, BSA was used in a mixture made of 20 µL of SOD (5.9 mg/mL), 10 µL of BSA (1.69% *w*/*v*) and 10 µL of GA (2.5% *v*/*v*) [61]. This mixture was dropped onto the surface electrode and stored at 4 °C before used and between measurements. The modified electrode was washed with purified water, before and after use.

## 3. Results

### 3.1. Optimization of Experimental Parameters

**Scheme 1:**
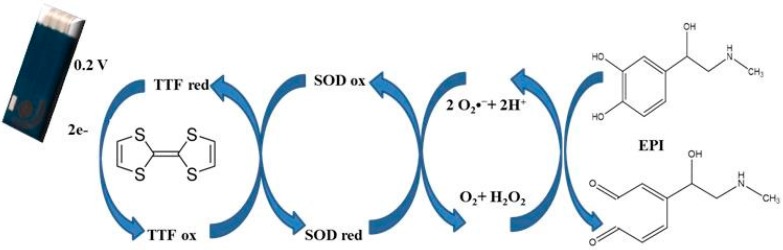
Oxidation of EPI on SOD/SPC_TTF_E.

EPI originates an amperometric signal at NPs/SPC_TTF_E with SOD enzyme immobilized (SOD/PdNPs/SPC_TTF_E), after which a steady-state current is reached. The presence of Al(III) ions produces SOD enzyme inhibition which causes a decrease in the EPI amperometric signal. Al(III) concentration influence inhibition process and can be quantitatively evaluated determining the difference between the steady state current in absence of Al(III), (I_0_), and the steady state current in the presence of Al(III), (I) namely Δ(I_0_-I).Accordingly, with the following working principle proposed in Scheme 1, a SOD based biosensor, with TTF incorporated in electrode ink, has been developed.

The parameter Δ(I_0_-I) depends on EPI concentration, applied potential (Eap) and pH solution. Therefore, an optimization of these variables was performed in order to ensure the quality of the results.

**Figure 2:**
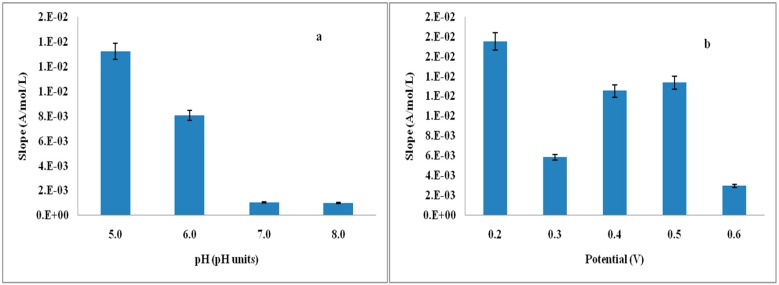
(**a**) Slope values of Al(III) calibration curves with pH; and (**b**) Slope value of Al(III) calibration curves with applied potential. [EPI] = 1.6 × 10^−4^ M; Britton Robinson buffer pH 5.0, Eap = +0.2 V vs. Ag/AgCl.

Because the dependence between Δ(I_0_-I) and Al(III) concentration is linear, substrate response was obtained from pH 5.0 to pH 8.0, and a pH of 5.0 was selected regarding substrate stability to autoxidation. In the same way, substrate response was obtained from +0.20 V to +0.60 V, and a potential of 0.2 V was selected driving substrate oxidation to epinephrinequinone [57]. Then, several aluminum inhibition calibration curves were performed at different potential and pH values and their slopes were compared, in order to obtain Al(III) inhibition effect with pH and Eap. Slope calibration curve with pH was calculated from pH 5.0 to pH 8.0. In the same way, slope calibration curve with potential was calculate from +0.20 V to +0.60 V. Higher slope values were obtained at pH 5.0 and Eap of +0.2 V, so these conditions were chosen to perform Al(III) inhibition calibration curves. Slopes of calibration curves with potential and pH are shown in Figure 2.

Findings indicated that substrate stability improved at low values of pH and potential; furthermore, if applied potentials were higher than +0.6 V, the electrodes showed erratic behavior. Since substrate response increases with concentration, a value of 1.6 × 10^−4^ M for EPI was chosen, as this concentration gives a proper sensibility, and a very stable signal with very low noise. Upper concentrations produced higher noise on amperometric recording of calibration curves. Under the selected conditions, the electrodes showed good performance. Calibration curves of Al(III) using SOD/AuNPs/SPC_TTF_Es, SOD/PtNPs/SPC_TTF_Es, SOD/PdNPs/SPC_TTF_Es, and SOD/RhNPs/SPC_TTF_Es were obtained under the optimized conditions. 

Preliminary experiments showed that modification of electrode surface with NPs increased the sensitivity of the biosensor; therefore, a thorough study of conditions of NPs deposition was carried out. AuNPs, PtNPs, PdNPs and RhNPs were deposited on electrodes surfaces according to methods described in the Experimental Section.

### 3.2. XRF and SEM for NPs/SPC_TTF_E Study METHOD A

Two different controlled potentials, +0.18 V and +0.3 V, were applied for 15 s to SPCEs in order to deposit NPs of every metal. X-ray fluorescence emission (XRF) spectra were obtained from surfaces of SPC_TTF_Es modified with the different type of NPs. Table 2 shows XRF percentage of elements deposited using indicated potentials. 

The plating of metals at +0.18 V for 15 s produced a higher percentage of Au, Pd and Rh. Pt deposited percentage was higher at +0.30 V. Since the Eap of +0.18 V applied for 15 s produced a higher percentage for Pd, Rh and Au, and the application of a deposition potential of +0.3 V did not deposit Pd or Au, conditions of Eap of +0.18 V and 15 s of method A were selected to deposit NPs of metals.

**Figure 3:**
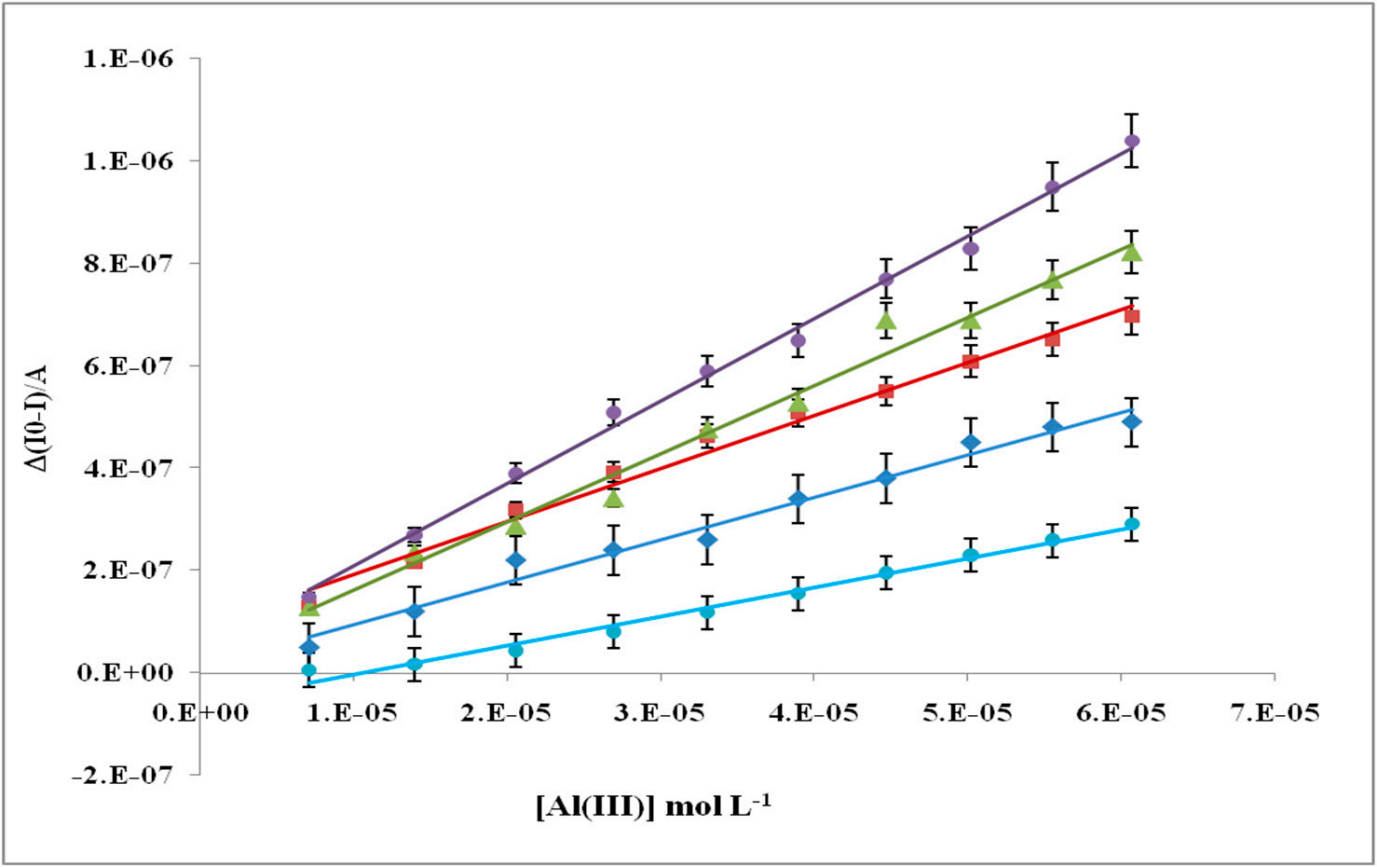
Calibration plots using: (●) SOD/AuNPs/SPC_TTF_Es; (■) SOD/PdNPs/SPC_TTF_Es; (▲) SOD/RhNPs/SPC_TTF_Es; (◆) SOD/PtNPs/SPC_TTF_Es and (●) SPC_TTF_Es performed with NPs/SPC_TTF_Es prepared by method A, [EPI] 1.6 × 10^−4^ M, Britton Robinson buffer pH 5.0, Eap = +0.2 V vs. Ag/AgCl.

The inhibition calibration curves for Al(III) are shown in Figure 3, where the lowest slope value corresponds to SPC_TTF_E without NPs deposited and the highest corresponds to SOD/AuNPs SPC_TTF_E. The other metal NPs modified SPC_TTF_E tested showed lower linear adjustment than SOD/AuNPs SPC_TTF_E. 

**Figure 4:**
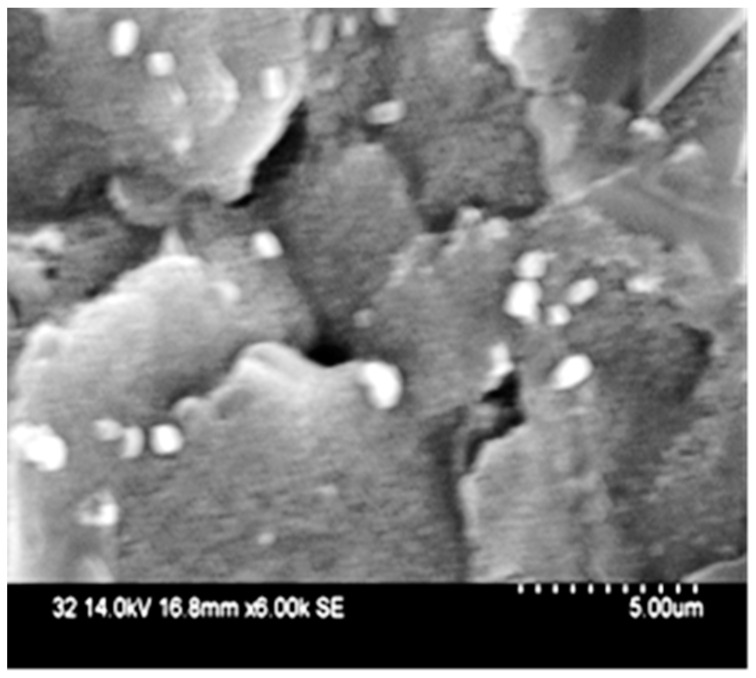
SEM image of AuNPs/SPC_TTF_E deposited at 0.18 V for 15 s.

SEM images of AuNPs obtained by method A deposited on SPC_TTF_E are presented in Figure 4.

### 3.3. XRF and SEM for NPs/SPC _TTF_Es Study METHOD B

**Figure 5:**
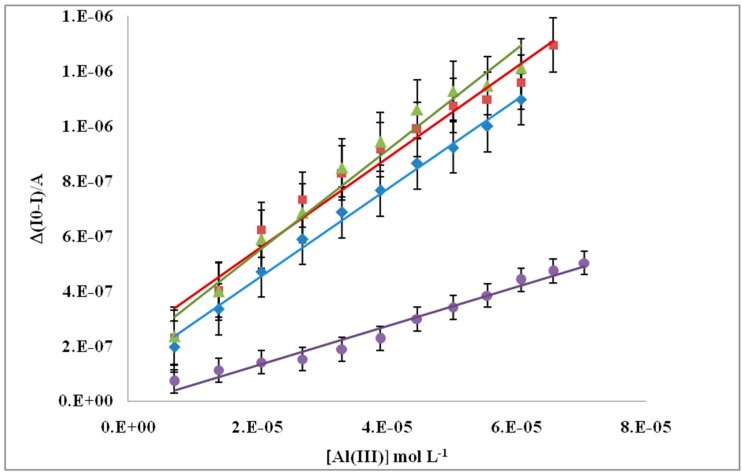
Calibration curves of Al(III) inhibition for (▲) SOD/RhNPs/SPC_TTF_Es; (■) SOD/PdNPs/SPC_TTF_Es; (◆) SOD/PtNPs/SPC_TTF_Es and (●) SOD/AuNPs/SPC_TTF_Es performed with NPs/SPC_TTF_Es prepared under CV1 condition, [EPI] 1.6 × 10^−4^ M, Britton Robinson buffer pH 5.0, Eap = +0.2 V vs. Ag/AgCl.

**Figure 6:**
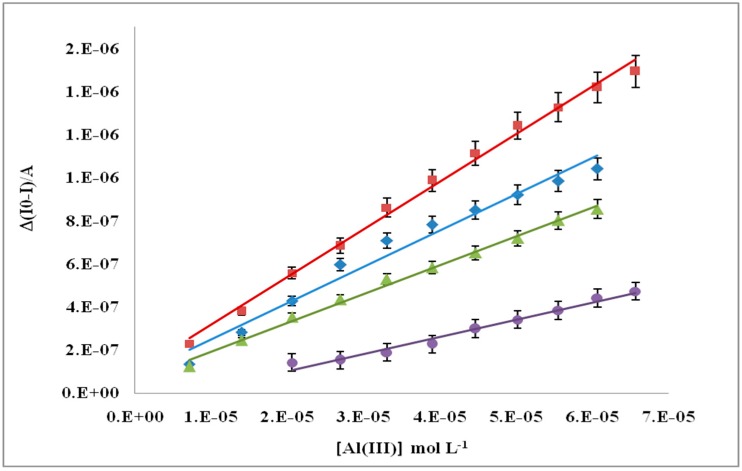
Calibration curves of Al(III) inhibition for (■) SOD/PdNPs/SPC_TTF_Es; (◆) SOD/PtNPs/SPC_TTF_Es; (▲) SOD/RhNPs/SPC_TTF_Es and (●) SOD/AuNPs/SPC_TTF_Es performed with NPs/SPC_TTF_Es prepared under CV2 conditions, [EPI] 1.6 × 10^−4^ M, Britton Robinson buffer pH 5.0; Eap = 0.2 V vs. Ag/AgCl.

**Figure 7:**
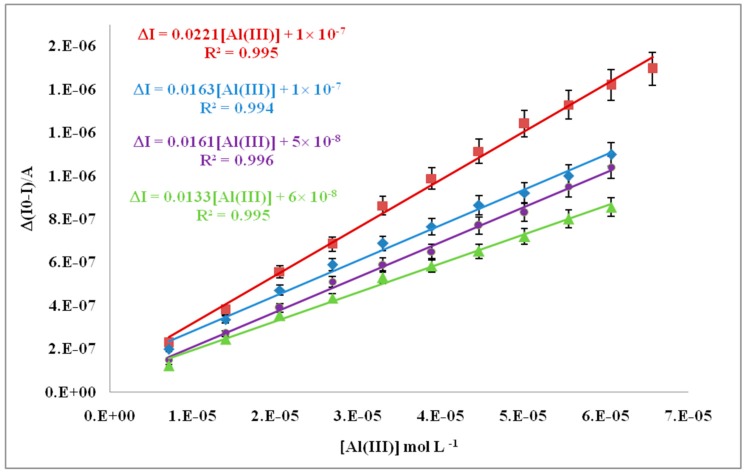
Calibration curves of Al(III) for (■) SOD/PdNPs/SPC_TTF_Es; (▲) SOD/RhNPs/SPC_TTF_Es prepared method B; CV2 conditions; (◆) SOD/PtNPs/SPC_TTF_Es, prepared by method B and CV1 conditions and (●) SOD/AuNPs/SPC_TTF_Es, prepared by method A, [EPI] = 1.6 × 10^−4^ M, Britton Robinson buffer pH 5.0, Eap = +0.2 V vs. Ag/AgCl.

**Figure 8:**
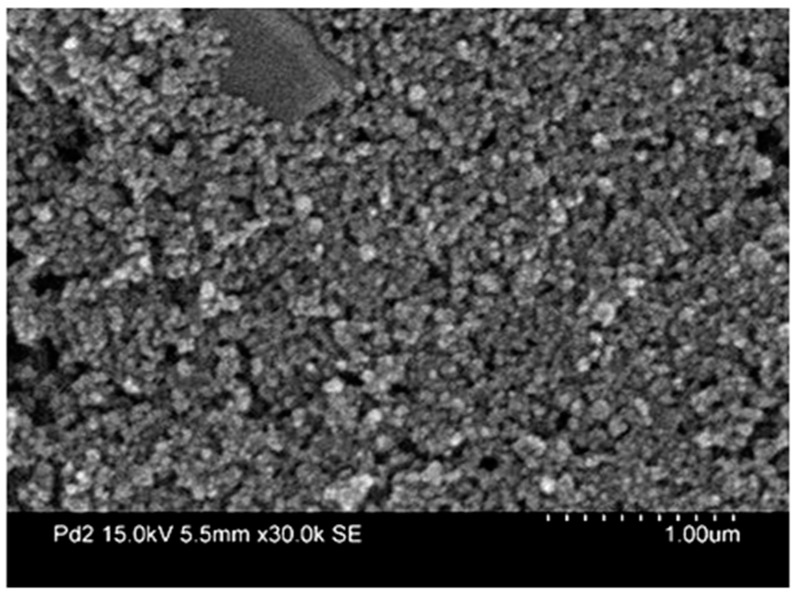
SEM image of PdNPs/SPC_TTF_E prepared by method B and CV2 conditions.

XRF percentagesforevery metal deposited with method B are shown in Table 1. AuNPs, PtNPs, PdNPs and RhNPs were deposited on SPC_TTF_Es according to method B and modified with immobilized SOD. SOD/SPCEs modified with metallic NPs showed the best linear adjustedAl(III) calibration curve at CV1 conditions for PtNPs and at CV2 conditions for PdNPs (Figure 5 and Figure 6). Regressions with the best linear fit performed by methods A and B showed that the highest slope corresponds to SOD/PdNPs/SPC_TTF_Es (Figure 7). SEM image of PdNPs/SPC_TTF_Es at CV2 conditions is shown in Figure 8, where it is observed that PdNPs are deposited in a regular form on SPC_TTF_Es for the CV2 conditions.

### 3.4. AFM Analysis of SPC_TTF_Es Prepared by Methods A and B

**Figure 9:**
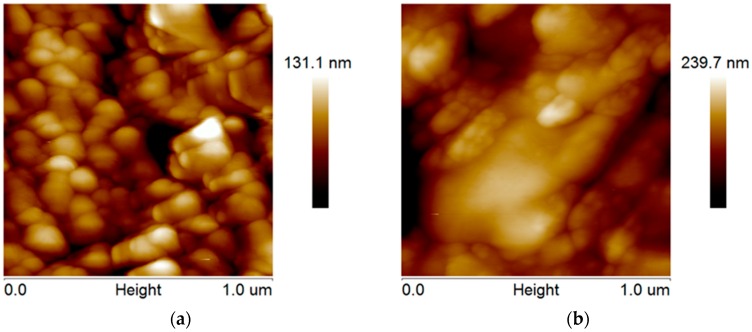
(**a**) AFM image for SPC_TTF_E control electrode; and (**b**) AFM image for AuNPs/SPC_TTF_E prepared by plating at 0.18 V for 15 s.

**Figure 10:**
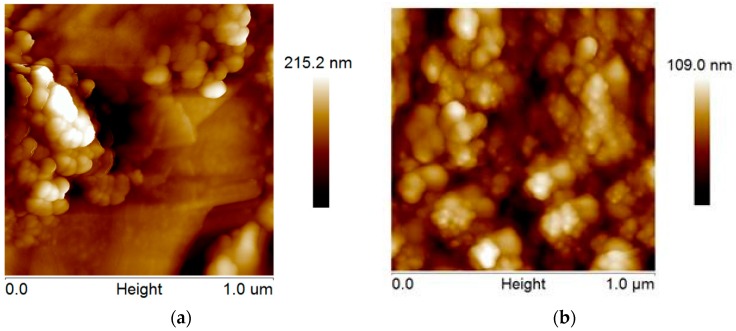
(**a**) AFM image of PdNPs/SPC_TTF_E prepared under method B andCV1conditions; and (**b**) AFM image of PdNPs/SPC_TTF_E prepared under method B and CV2 conditions.

AuNPs deposit on SPC_TTF_E increases roughness of SPC_TTF_E compared with control electrode, as can be observed in AFM images of the surfaces of AuNPs/SPC_TTF_Es obtained by deposition of AuNPs at 0.18 V for 15 s (Figure 9). The highest slope presented for SOD/AuNPs/SPC_TTF_E using method A is afforded by lower AFM parameters of AuNPs at 0.18 V for 15 s, than when the CV1or CV2 conditions are used. AFM images of PdNPs/SPC_TTF_Es obtained by deposition of PdNPs at CV1 and CV2 conditions are shown in Figure 10. Analysis of AFM parameters confirmed that metal deposited was nanometric size. Table 3 shows the most important parameters of NPs/SPC_TTF_Es and SPC_TTF_Es control electrode obtained through tapping mode and Roughness Kurtosis (RKu) and Skewness (RSk) coefficients. PdNPs/SPC_TTF_Es, obtained using CV2 conditions, showed lower Roughness Average (RA), Roughness Mean Square (RMS) and height of the highest peak above mean line in the profile (Rmax) than other SPC_TTF_Es modified with metallic NPs, indicating that PdNPs/SPC_TTF_Es prepared by CV2 condition present a more homogeneous surface [62]. AFM image of PdNPs prepared by CV2 method showed lower values of RA andRMS when compared tosurface prepared by CV1 method.

It was observed that PdNPs/SPC_TTF_Es modified by means of method B and CV2 conditions showed lower AFM values than the other NPs/SPC_TTF_Es. Then, it was decided to analyze the response of EPI at SOD/NPs/SPC_TTF_Es in presence of aluminum.

### 3.5. Inhibition Behavior of Al(III) on SOD Enzyme

**Figure 11:**
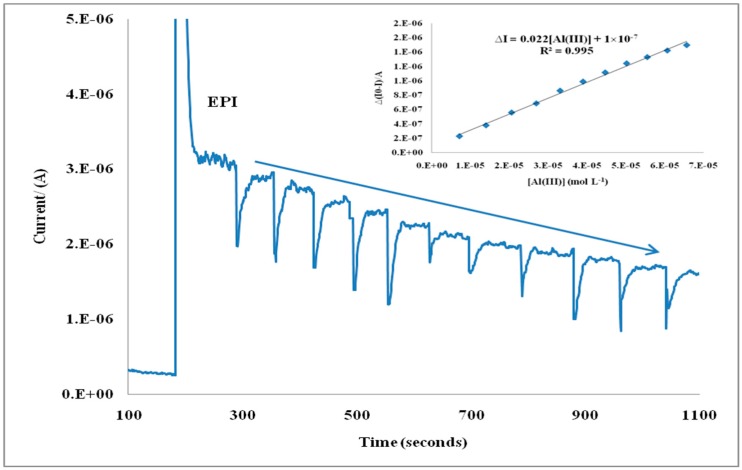
Amperometric recording performed with SOD/PdNPs/SPC_TTF_E, prepared under method B and CV2 conditions, [EPI] = 1.6 × 10^−4^ M and consecutive additions of 100 µL of Al(III) 3.7 × 10^−4^ M into the cell, Britton Robinson buffer pH 5.0, Eap = +0.20 V vs. Ag/AgCl. Inset: Calibration curve corresponding to this amperogram.

Michaelis Menten Km apparent constants were estimated by Lineweaver–Burk plot. It was obtained in presence and absence of Al(III) with SPC_TTF_Es modified with AuNPs, PtNPs, RhNPs and PdNPs. Modified electrodes were prepared under the best conditions for each NP deposition method used, namely 0.18 V for method A and CV2 conditions for method B. Figure 11 shows amperometric recording of SOD/PdNPs/SPC_TTF_Es (obtained by method B and CV2 conditions).

Km apparent values of modified electrodes are shown in Table 4.

### 3.6. Validation of SOD/PdNPs/_TTF_/SPCE Based Biosensor

SOD/PdNPs/SPC_TTF_Es were selected to perform validation of the developed biosensor trough estimation of their performance parameters.

#### 3.6.1. Limit of Detection

The limit of detection under the optimum working conditions (2.0 ± 0.2 μM) was calculated from the standard deviation (Sy/x) of five Al(III) inhibition calibration curves according to the criteria 3 Sy/x [63], and its RSD was 7.9%. Analogous to LOD, quantification limit (LOQ) was estimated under optimal conditions from the standard deviation of five Al(III)inhibition calibration curves using the criteria 10 Sy/x, and its value was 6.7 ± 0.5 μM, with a RSD of 7.9%. 

#### 3.6.2. Precision

This parameter is usually calculated in terms of reproducibility and repeatability. Repeatability was assessed using the same electrode surface. In this way, successive calibrations for Al(III) were tested with SOD/PdNPs/SPC_TTF_Es prepared under CV2 conditions. The electrodes were conditioned in a Britton Robinson buffer solution, pH 5.0, stirring for 5 min between experiments. The RSD obtained for the slopes of the first two graphs was 5.1%, but, in the third measurement, a decrease in the biosensor sensitivity and a RSD increase, reaching 15%, were observed. Because the electrodes are disposables, the reproducibility is a better estimate of performance. Likewise, the reproducibility of the amperometric signal was checked using the slopes of five regression lines carried out with different electrode surfaces, RSD slope value estimated was 7.0%. 

#### 3.6.3. Accuracy

The accuracy of the developed method was tested by a recovery study in which a known amount of Al(III) standard reference material (SRM), SRM High Purity Standards solution (Lot Number 1121015, (1000 ± 3) mg·L^−1^) was spiked to a buffer solution. 

The aluminum average concentration quantified by the developed procedure, 1038 ± 50 mg·L^−1^ (n = 5; α = 0.05), matches the certified value of the sample considering the associated uncertainty. The mean recovery percentage obtained was (103.8 ± 4.8)%. Recovery values are shown in Table 5. SRM was spiked to tap water replicates, SRM aluminum average concentration found was 1005 ± 34 mg·L^−1^ (n = 3; α = 0.05).Mean recovery percentage obtained was (100.5 ± 3.4)%. Recovery values are shown in Table 6. 

### 3.7. Study of Interferences on SOD/PdNPs/SPC_TTF_E Biosensors

**Figure 12:**
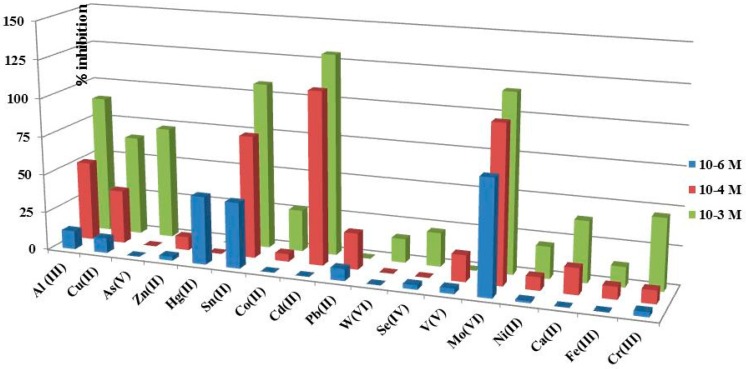
Inhibition percentage of current at SOD/PdNPs/SPC_TTF_E, (method B and CV2 conditions) in presence of: Al(III), Cu(II), As(V), Zn(II), Hg(II), Sn(II), Co(II), Cd(II), Pb(II), W(VI), Se(IV), V(V), Mo(VI), Ni(II), Ca(II), Fe(III) and Cr(III) at three concentration levels; [EPI] = 1.6 × 10^−4^ M; Britton Robinson buffer pH 5.0, Eap = +0.2 V vs. Ag/AgCl.

Interference study was performed comparing the percentage of inhibition showed by the developed SOD based biosensor in the presence of aluminum and other foreign ions. Three concentration levels of possible interfering ions, namely 1 × 10^−3^ M, 1 × 10^−4^ M, and 1 × 10^−6^ M, were tested. Regarding Al(III), LOD value obtained for SOD/PdNPs/SPC_TTF_E is meaningful at 1 × 10^−4^ M. As can be seen in Figure 12, the highest interference effect was found for Sn(II), Cd(II) and Mo(VI) for concentrations tested; however, these cations should usually not be present in water.

## 4. Discussion

SOD biosensor was developed looking for effect of metallic NPs on sensibility of slopes of Al(III) calibration curves. One initial hypothesis was that NPs generation methodology influence sensitivity of biosensor. For these reasons, two different methodologies to deposit NPs were tested, direct deposit at 0.18 V produced the highest slope for AuNPs, CV2 deposit methodology produced the highest slope for PdNPs, followed for RhNPs andCV1 deposit methodology produced the highest slope for PtNPs. Criteria used for slope selection were linearity and sensibility. Regarding all optimum slopes values obtained with SOD/NPs/SPC_TTF_Es biosensors under the two methodologies, the highest slope value was obtained for SOD/PdNPs/SPC_TTF_Es based biosensor. NPs’ physical characteristics are also modified by deposit methodologies.Although SEM were performed on every NPs/SPC_TTF_Es prepared by means of the above-mentioned methodologies and NPs were visualized, AFM is a more appropriate instrument for NPs/SPC_TTF_Es surface characterization. AuNPs/SPCTTFEs prepared by method A showed lower AFM parameters, namely RA, RMS and Rmax, than CV deposition methodologies. PdNPs/SPC_TTF_Es prepared by CV2 showed lower AFM parameters than PdNPs/SPC_TTF_Es prepared by CV1. Although PtNPs/SPC_TTF_Es prepared by CV2/CV1 showed similar AFM values and slopes, best linearity was obtained for CV2 condition. RhNPs/SPC_TTF_Es prepared under CV2 condition showed much better linearity than CV1. All RKu values are near 3, providing evidence that the obtained values are closer to a normal distribution, and the surface is named Mesokurtic, for kurtosis minor 3, surface is flat and called Platykurtic. When kurtosis is greater than 3, surface owns more peaks than valleys. RSk measures the profile of symmetry about mean line. If the height distribution is asymmetrical and the surface has more peaks than valleys, skewness is positive, while in the opposite case, skewness is negative.

SOD/NPs/SPC_TTF_Es based biosensor was based on Al(III) inhibition of SOD, and Km inhibition were estimated for SOD/NPs/SPC_TTF_Es under method A andCV2 condition. It was established that Al(III) exerts its inhibitory action at low concentration. Inhibitory effect for SOD/NPs/SPC_TTF_Es was confirmed by means of Km app values with Al(III) increasing concentrations. This fact is in accordance with theoretical considerations regarding enzymatic behavior in presence of inhibitors. However, the last measurement for SOD/RhNPs/SPC_TTF_Es, displayed an unusual behavior. 

SOD/PdNPs/SPC_TTF_Es were selected to perform validation of the developed biosensor. The selection was based on the Al(III) inhibition calibration curves of SOD enzyme, that clearly showed higher sensibility by modifying SPC_TTF_Es with PdNPs prepared under method B and CV2 conditions than the other metallic NPs. Al(III) inhibition on SOD/NPs/SPC_TTF_Es could be used with analytical purposes, but at first it is necessary to perform developed biosensor validation. This goal was achievedby performance parameters estimation. Precision was established through reproducibility of calibration curves slope, as SPC_TTF_Es are disposable, and this reproducibility is a good precision estimate. LOD and LOQ values allowed quantification of low Al(III) concentrations. Recovery percentage of certified SRM afforded accuracy of SOD/NPs/SPC_TTF_Es based biosensor and indicated that developed SOD/PdNPs/SPC_TT_Es biosensor can be applied to Al(III) determination in aqueous solutions.Validation results suggest that the fabrication procedure of the SOD/PdNPs/SPC_TTF_Es based biosensor is reliable and allows reproducible amperometric responses to be obtained with different electrodes constructed using the method described in this work.

A weakness of biosensor SOD/PdNPs/SPC_TTF_Es is its response to interfering ions, but these toxic ions should not be naturally present in water. Ca(II) and Fe(III) usually found in water do not interfere. Al(III) showed inhibition on SOD enzyme at all tested concentrations.

## 5. Conclusions

A novel amperometric biosensor based on SOD/PdNPs/C_TTF_/SPCEs was developed, validated and applied to Al(III) determination in aqueous matrixes. The biosensor was based on inhibitory effect of Al(III) on SOD enzyme and presents fast response, very good reproducibility, stability, and low LOD. Michaelis Menten constants were calculated from Lineweaver–Burk plots and showed increasing values with Al(III) concentration in accordance with theory of enzymatic inhibition.

SOD enzyme immobilization was easily and rapidly achieved by crosslinking using glutaraldehyde and allowed obtaining a good reproducibility value of biosensor. 

Modification of SPC_TTF_Es with different types of NPs improves biosensor performance. A study of electrolytic generation conditions of NPs of Au, Pt, Rh and Pd onto SPC_TTF_ E surface was carried out and results showed that SPC_TTF_Es modified with PdNPs by means of cyclic voltammetry under method B and CV2 conditions (delay time 120 s, step potential, 0.025 V, scan rate 0.1 V/s) gave a higher sensibility on amperometric inhibition of Al(III) calibration curves. 

SEM images showed presence of the metallic NPs deposited on SPC_TTF_Es. XRF study was conducted to evaluate percentages of every metal deposited on SPC_TTF_Es. In addition, AFM study showed roughness, characteristic of SPC_TTF_Es and NPs/SPC_TTF_Es surfaces and provided useful information about morphology and surface homogeneity. It was also found that PdNPs/SPC_TTF_Es deposited by method B and CV2 conditions had lower RA, RSM and Rmax than the others metallic NPs. 

Biosensor validation was performed under optimized conditions: pH 5.0, applied potential of 0.2 V and a concentration of EPI of 1.6 × 10^−4^ M. The recovery value obtained using certified material, supported the feasibility of SOD/PdNPs/SPC_TTF_Es based biosensor for Al(III) determination. 

Developed biosensor presents LOD similar to other developed Al(III) biosensors, but it has the advantage of using a lower applied potential of only +0.2 V. The possibility of using this biosensor at low potentials results in a muchhigher selectivity compared with the others Al(III) biosensors. 

## Figures and Tables

**Table 1:** SOD based biosensors applied to samples of biological interest.

Technique	Electrode	Potential	Modification	Range	LOD	Sample/Analite	Reference
CV ^1^	SPCE	−0.8–+0.8 V	Pyrrole/SAMs	0.5 × 10^−9^–5 M	0.5 × 10^−9^ M	cultured human keratinocytes NO_2_^-^	[49]
Amperometry	CFME ^2^	+0.25 V	cysteine/AuNPs	(13–104) × 10^−9^ M	-	O_2_•^−^	[50]
Amperometry	SPCE	−0.1 V	porous Pt-Pd/nafion	(16–1536) × 10^−6^ M	0.13 × 10^−6^ M	Cell culture medium/O_2_•^−^	[51]
CV ^1^ Chronoamperometry	Gold electrode	−0.2–+0.5 V	Au/Cys/SOD ^9^, Au/GNP/Cys/SOD ^10^ and Au/GNP/Cys/SOD/Chit ^11^	0.5–4 Gy	0.03 × 10^−6^ M	thallium 201/water	[52]
Amperometry	GC CFME ^2^	0.2 V	NTA/HT ^7^	10^−^^7^–10^−^^4^ M	21 × 10^−9^ M	brain tissue/O_2_•^−^	[53]
CV ^1^	Carbon paste electrode Electrochem	0–0.3 V	cytochrome c in solution and Fe(III)-protoporphyrin immobilized	(1–6) × 10^−3^ M	0.3 × 10^−3^ M	Xantine	[54]
	System Carbon paste electrode		Protoporphyrin and cytochrome both immobilized	(1–8) × 10^−3^ M	0.2 × 10^−3^ M		
Cronoamperometry	Composite electrode	−0.3 V	PtPd-PDARGO ^6^	(0.016–0.24) × 10^−3^ M	2 × 10^−6^ M	DMEM ^5^/O_2_•^−^	[55]
Amperometry	GC ^8^	−0.3 V	MWCNT ^4^	(0.01–0.3) × 10^−3^ M	1 × 10^−6^ M	Wines, berry juice/O_2_•^−^	[56]
			PEDOT ^3^				
Amperometry	SPC_TTF_E ^12^	0.2 V	SOD/PdNP ^13^	(1.0–60) × 10^−5^ M	2 × 10^−6^ M	Al(III)	This article
						water	
						samples	

^1^ Cyclic voltammetry; ^2^ carbon fiber microelectrode; ^3^ poly(3,4-ethylenedioxythiophene); ^4^ multiwalled carbon nanotubes, ^5^ Dulbecco’s modified Eagle’s medium; ^6^ PtPd poly dopamine reduced graphene oxide; ^7^ Nitrilotriacetic acid/histidine-tag; ^8^ glassy carbon; ^9^ gold, cysteine, super oxido dismutase; ^10^ gold, gold nanoparticles cysteine, super oxido dismutase; ^11^ gold, gold nanoparticles cysteine, super oxido dismutase, chitosane; ^12^ screen printed carbon TTF5% electrode; ^13^ super oxide dismutase, palladium nanoparticles.

**Table 2:** XRF percentage of element deposited on SPC_TTF_Es by method A and B.

	Method A		Method B	
Element	XRF%	XRF%	XRF%	XRF%
	(+0.18 V, 15 s)	(+0.30 V, 15 s)	CV1	CV2
Pd	0.136	0.00	0.557	0.632
Pt	0.223	1.48	2.74	2.71
Rh	0.693	0.380	4.49	2.95
Au	1.42	-	1.87	2.23

**Table 3:** AFM parameters of modified SPC_TTF_Es with metallic NPs.

NPs/SPC_TTF_Es	Method/Conditions	RA (nm)	RMS (nm)	Rmax (nm)	RKu	RSk
SPC_TTF_E	-	16.8	21.5	131	3.53	−0.183
AuNPs/SPC_TTF_E	A/0.18 V	31.3	39.2	218	3.34	−0.537
	B/CV1	33.8	41.4	234	2.62	0.126
	B/CV2	34.6	44.5	303	3.19	−00292
PdNPs/SPC_TTF_E	B/CV1	24.8	34.6	216	4.72	0.0604
	B/CV2	14.7	18.2	106	2.69	0.0939
PtNPs/SPC_TTF_E	B/CV1	106	140	864	3.63	0.157
	B/CV2	106	140	864	3.60	0.141
RhNPs/SPC_TTF_E	B/CV1	22.0	28.1	173	3.30	−0.0733
	B/CV2	25.6	33.9	204	3.65	−0.189

**Table 4:** Km apparent values for SPC_TTF_Es modified with AuNPs, PtNPs, RhNPs, and PdNPs.

Km Apparent (M)	SOD/AuNPs/SPC_TTF_Es	SOD/PtNPs/SPC_TTF_Es	SOD/PdNPs/SPC_TTF_Es	SOD/RhNPs/SPC_TTF_Es
Method A 0.18 V				
Without Al	(7.8 ± 0.3) × 10^−4^	(1.2 ± 0.3) × 10^−4^	(1.5 ± 0.4) × 10^−3^	(3.3 ± 0.2) × 10^−3^
Al(III) 7.25 × 10^−6^ M	(1.3 ± 0.1) × 10^−3^	(1.4 ± 0.3) × 10^−2^	(1.0 ± 0.1) × 10^−2^	(1.4 ± 0.1) × 10^−2^
Al(III) 2.18 × 10^−5^ M	(3.7 ± 0.3) × 10^−3^	(1.8 ± 0.6) × 10^−2^	(1.3 ± 0.1) × 10^−2^	-
Method B and CV2 conditions				
Without Al	(1.5 ± 0.2) × 10^−3^	(5.8 ± 0.3) × 10^−4^	(1.5 ±0.3) × 10^−3^	(1.2 ± 0.1) × 10^−4^
Al(III) 7.25 × 10^−6^ M	(2.7 ± 0.4) × 10^−3^	(1.3 ± 0.4) × 10^−3^	(5.0 ±0.4) × 10^−3^	(6.4 ± 0.5) × 10^−3^
Al(III) 2.18 × 10^−5^ M	(3.05 ± 0.5) × 10^−3^	(3.0 ± 0.8) × 10^−3^	(1.2 ±0.3) × 10^−2^	(5.4 ± 0.2) × 10^−3^

**Table 5:** Recovery of Al(III) SRM (1000 mg/L ± 3 mg/L) spiked to buffer solution.

Added SRM	Found SRM	Found SRM	SRM	Recovery
(M)	(M)	(mg/L)	(mg/L)	-
1.30 × 10^−5^	1.235 × 10^−5^	9.50	950	95.0
-	1.366 × 10^−5^	10.51	1051	105.1
-	1.370 × 10^−5^	10.51	1051	105.3
-	1.374 × 10^−5^	10.58	1058	105.8
-	1.402 × 10^−5^	10.79	1079	107.9
-	-	Mean	1038	103.8
-	-	SD	50.3	5.0
-	-	RSD	4.8	4.8

**Table 6:** Recovery of SRM (1000 mg/L ± 3 mg/L) spiked to tap water.

Added SRM (mg/L)	Found SRM (mg/L)	SRM (mg/L)	SRM (mg/L)	Recovery (%)
0.170	0.180	10.44	1044	104.0
	0.171	9.90	990	99.0
	0.169	9.82	982	98.2
-	-	Mean	1005	100.5
-	-	SD	34	3.4
-	-	RSD	3.4	3.4
